# Sepsis Induces a Dysregulated Neutrophil Phenotype That Is Associated with Increased Mortality

**DOI:** 10.1155/2018/4065362

**Published:** 2018-04-11

**Authors:** Jaimin M. Patel, Elizabeth Sapey, Dhruv Parekh, Aaron Scott, Davinder Dosanjh, Fang Gao, David R. Thickett

**Affiliations:** Institute of Inflammation and Ageing, University of Birmingham, Birmingham, UK

## Abstract

**Background:**

Neutrophil dysfunction in sepsis has been implicated in the pathogenesis of multiorgan failure; however, the role of neutrophil extracellular traps (NETs) remains uncertain. We aimed to determine the sequential changes in ex vivo NETosis and its relationship with mortality in patients with sepsis and severe sepsis.

**Methods:**

This was a prospective observational cohort study enrolling 21 healthy age-matched controls and 39 sepsis and 60 severe sepsis patients from acute admissions to two UK hospitals. Patients had sequential bloods for the ex vivo assessment of NETosis in response to phorbol-myristate acetate (PMA) using a fluorometric technique and chemotaxis using time-lapse video microscopy. Continuous data was tested for normality, with appropriate parametric and nonparametric tests, whilst categorical data was analysed using a chi-squared test. Correlations were performed using Spearman's rho.

**Results:**

Ex vivo NETosis was reduced in patients with severe sepsis, compared to patients with sepsis and controls (*p* = 0.002). PMA NETosis from patients with septic shock was reduced further (*p* < 0.001) compared to controls. The degree of metabolic acidosis correlated with reduced NETosis (*p* < 0.001), and this was replicated when neutrophils from healthy donors were incubated in acidotic media. Reduced NETosis at baseline was associated with an increased 30-day (*p* = 0.002) and 90-day mortality (*p* = 0.014) in sepsis patients. These findings were accompanied by defects in neutrophil migration and delayed apoptosis. Resolution of sepsis was not associated with the return to baseline levels of NETosis or migration.

**Conclusions:**

Sepsis induces significant changes in neutrophil function with the degree of dysfunction corresponding to the severity of the septic insult which persists beyond physiological recovery from sepsis. The changes induced lead to the failure to effectively contain and eliminate the invading pathogens and contribute to sepsis-induced immunosuppression. For the first time, we demonstrate that reduced ex vivo NETosis is associated with poorer outcomes from sepsis.

## 1. Introduction

The incidence of sepsis is continuing to rise and accounts for approximately 215,000 deaths per year in the United States of America (USA) [1]. The management of sepsis places a large financial burden on health care systems with conservative estimates suggesting that the USA spends $17 billion treating sepsis annually [1–3].

Sepsis is a complex syndrome that has been defined as a life-threatening immune response to infection [4]. However, the pathogen load and its virulence and the subsequent host characteristics determine the extent and nature of this response [2, 5]. Neutrophils are one of the first lines of defense against invading pathogens and are responsible for containing and eliminating invading pathogens [6, 7]. Neutrophils are multifaceted innate immune cells that also modulate the inflammatory response and initiate the adaptive immune responses to sepsis via the release of cytokines. It is this coordinated response that maintains immune homeostasis [8].

In sepsis, there is a dysregulated immune response with activated circulating neutrophils releasing cytokines and reactive oxygen species (ROS) at sites distal to the infectious focus leading to multiorgan failure [7]. Additionally, neutrophils have been shown to demonstrate immunosuppressive phenotypes, with immaturity and altered chemokine expression responsible for some of these alterations [8]. This has pertinence in sepsis, with sepsis-induced immunosuppression being recognized as a clinical syndrome in survivors of sepsis, who have an increased susceptibility to nosocomial infections, frequent hospital readmissions, and subsequently increased late mortality [7, 9–11]. Recently, we described a reduction in systemic neutrophil migratory accuracy in lower respiratory tract infections, pneumonia, and a mild, ward-based pneumonia-associated sepsis cohort with evidence of prolonged migratory dysfunction after the septic event [12]. It is unclear whether reduced neutrophil migratory accuracy might also be a feature of a more severe sepsis cohort, how this might change over time, and whether other facets of neutrophil function might also be affected.

Following activation, triggered either by frustrated phagocytosis or sustained inflammation, neutrophils release neutrophil extracellular traps (NETs), whereby nuclear DNA laden with histones and granular contents are liberated into the extracellular space, which trap and kill extracellular bacteria [13, 14]. The exact role of NETs in sepsis remains uncertain. Studies in murine models of sepsis have shown that an inability to generate NETs in response to infection leads to increased severity of insult and death [14–17]. Additionally, similar studies have demonstrated that NET formation in intravascular beds, such as liver sinusoids, and within the alveoli causes endothelial damage and leads to patterns of organ dysfunction which are the hallmark of sepsis [18–22]. However, studies linking poor clinical outcomes with neutrophil functions, and the production of NETs in patients with sepsis over time, are lacking.

One of the hallmarks of sepsis is tissue hypoperfusion and tissue hypoxia leading to a switch to anaerobic glycolysis and the development of a metabolic lactic acidosis, with the degree of metabolic acidosis often used as a surrogate marker for severity of the inflammatory/infectious insult [4]. The role of acidosis in dysregulated neutrophil function in sepsis is poorly understood.

We hypothesized that sepsis severity (clinical and biochemical) would be associated with impairment of neutrophil functions, and in particular NETs, with worse clinical outcomes being seen in patients with most evidence of innate immunoparesis. Additionally, we hypothesized that the severity of acidosis would also be related to NET suppression. To test this, we aimed to assess neutrophil functions in patients hospitalized with sepsis and relate this to markers of sepsis severity and short and long-term clinical outcomes.

## 2. Materials and Methods

Adult patients admitted to a hospital with sepsis were screened and enrolled within 48 hours of admission to hospital. Sepsis, severe sepsis, and septic shock were defined based on the criteria used by the Surviving Sepsis Campaign Guidelines of 2008 (online Supplementary Materials available here) [23].

Patients were recruited from the University Hospital Birmingham and the Heart of England NHS Foundation Trust between September 2012 and June 2014 with 1-year follow-up completed in June 2015. Patients had blood drawn on enrollment and where possible on day 4 and day 7. Healthy aged controls (≥60 years with no systemic disease or only mild systemic disease; stage 1 hypertension/mild asthma) were also recruited.

This study was carried out per the Declaration of Helsinki, and all patients and healthy controls were consented. Patients were consented, and where not possible, assent was gained from their next of kin or physician. These studies received appropriate ethical approvals (Regional Ethics Committee references: 11/SC/0356 and 11/YH/0270). This research was undertaken prior to the publication of the new sepsis definitions of 2015. The new definitions identify a population at high risk of in-hospital mortality, but exclude milder infections, which this study sought to include [4].

### 2.1. Isolation of Neutrophils

Neutrophils were isolated on a Percoll (pH 8.5–9.5; Sigma-Aldrich, UK) density gradient as previously described [24, 25]. The neutrophils (95% pure and 97% viable by trypan blue exclusion) were resuspended in RPMI 1640 (Sigma-Aldrich).

### 2.2. Neutrophil Extracellular Trap Assay

Freshly isolated neutrophils (1 × 10^5^ cells) were stimulated to generate NETs by incubating them within the control media (RPMI 1640 supplemented with glutamine, penicillin, and streptomycin-GPS; Sigma-Aldrich) or in the positive control 25 nM phorbol-myristate acetate (PMA, Sigma-Aldrich) as previously described using a fluorometric technique [26]. NET production was measured as arbitrary fluorescent units (AFUs).

### 2.3. Neutrophil Migration Assay

Migration was assessed using an Insall Chamber (Weber Scientific International Ltd., UK) as described previously [27, 28]. Migration was assessed towards a vehicle control (RPMI 1640), 100 nM interleukin-8 (CXCL-8) (R&D Systems, UK). Time-lapse video microscopy was used to capture neutrophil migration and analysed using ImageJ vector analysis software (Wayne Rasband, Bethesda) to calculate chemotaxis (directional migration) [12].

### 2.4. Cell-Free DNA Measurement

Cell-free DNA (cf-DNA) levels were measured from stored plasma samples using a fluorometric assay and SYTOX Green Dye (Life Technologies, UK) as previously described [29]. Values are represented as ng/ml of cf-DNA.

### 2.5. Neutrophil Apoptosis Assay

Freshly isolated neutrophils (1 × 10^5^ cells) were suspended in RPMI 1640 supplemented with GPS. Apoptosis experiments were performed by flow cytometry (CyaN_ADP_; Beckham Coulter) on isolation, at 4 hours and 24 hours.

To account for variations in baseline rates of apoptosis, the percentage change in apoptosis at 4 hours and 24 hours was calculated.

### 2.6. Statistical Analysis

Statistical analysis was performed using GraphPad Prism Version 6 (La Jolla, USA). Continuous data was tested for normality using a Shapiro-Wilk test. Parametric data are represented as mean ± SEM and were analysed using a Student's *t*-test (two independent samples) or a one-way analysis of variance (ANOVA) test with a post hoc Bonferroni test (more than 2 groups). Nonparametric data are represented as median (IQR) and were analysed with a Mann–Whitney *U* test (two independent samples) or a Kruskal-Wallis test (more than 2 groups) with Dunn's post hoc test. A Pearson or Spearman correlation was used for parametric and nonparametric data, respectively. Categorical data was analysed using Fisher's exact test for two variables and a chi-squared (*χ* ^2^) test used when greater than two groups were analysed. All tests were two-tailed with results considered significant if *p* < 0.05.

## 3. Results

### 3.1. Participant Characteristics

**Figure 1:**
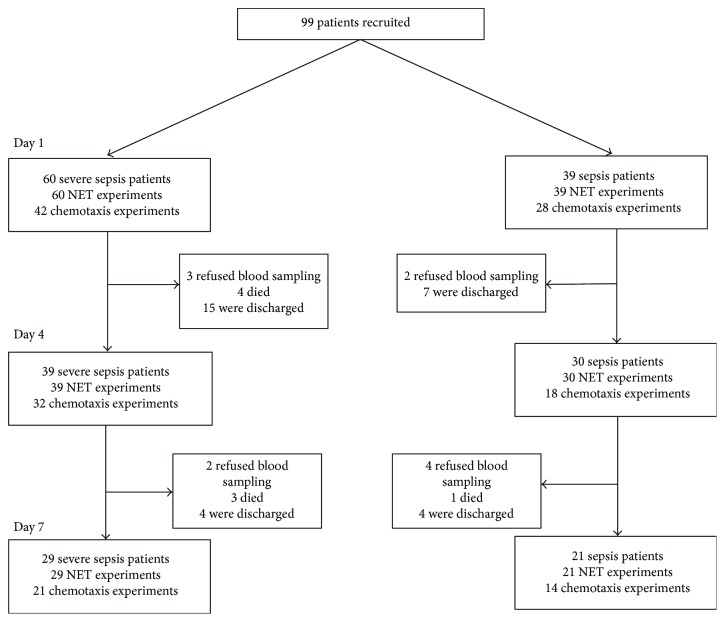
Consort diagram of experiments. Modified consort diagram of the number of experiments performed at the various time points during the study. The reducing number of experiments performed between the groups was due to either deaths, discharges from hospital, or refusals of blood sampling from patients.

39 patients with sepsis, 60 patients with severe sepsis, and 21 age-matched healthy controls were recruited. Predictably, patients with sepsis had significantly greater comorbidities and were taking more medications than healthy controls were. However, the patients recruited into the sepsis and severe sepsis cohorts were well matched with no significant differences for age, sex, preexisting diseases, or medications being taken. The demographics of enrolled participants are shown in Table 1 with an experimental consort diagram shown in Figure 1.

### 3.2. Severe Sepsis, but Not Sepsis, Suppresses NET Production

**Figure 2:**
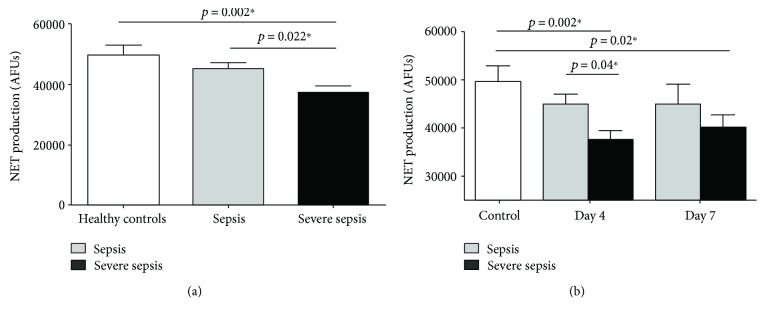
The ex vivo production of NETs in healthy controls and patients with sepsis and severe sepsis. (a) NETosis from 21 healthy controls, 39 patients with sepsis, and 60 patients with severe sepsis following stimulation with 25 nM PMA for 4 hours ex vivo. An ANOVA between all 3 groups showed a significant difference where *p* = 0.002, with ∗ representing the significant differences in a post hoc Tukey's test. (b) NETosis from 21 healthy controls, 30 patients with sepsis, and 39 patients with severe sepsis on day 4 following stimulation with 25 nM PMA for 4 hours ex vivo. An ANOVA between all 3 groups showed a significant difference (*p* = 0.001), with ∗ representing significant differences in a post hoc Tukey's test. On day 7, there are 21 sepsis patients and 29 severe sepsis patients. There was a significant difference between healthy controls and patients with severe sepsis^∗∗^.

Ex vivo NETosis in healthy controls were compared to patients diagnosed with sepsis and severe sepsis on admission to hospital. No differences were seen in baseline NETosis in unstimulated neutrophils between groups (8219 ± 2796 AFUs versus 7219 ± 4685 AFUs versus 7191 ± 5141 AFUs; ANOVA, *p* = 0.701). PMA-stimulated neutrophils from healthy controls and from patients with sepsis generate similar levels of NETs (healthy controls: 49659 ± 3285 AFUs versus sepsis: 45304 ± 1777 AFUs; Student's *t*-test, *p* = 0.207; Figure 2(a)). However, in neutrophils from patients with severe sepsis, NETosis (37181 ± 2204 AFUs) was significantly abrogated compared to both healthy controls (49659 ± 3285 AFUs) and patients with sepsis (45304 ± 1777 AFUs; ANOVA, *p* = 0.002; see Figure 2(a)). In a subgroup of patients with severe sepsis that had septic shock (*N* = 13), NETosis was further attenuated compared to the healthy controls (23785 ± 2853 AFUs versus 49,659 ± 3285 AFUs; Student's *t*-test, *p* < 0.001) and patients with sepsis (23785 ± 2853 AFUs versus 45304 ± 1777 AFUs, *p* < 0.001).

### 3.3. Persistent Attenuation of NETosis over Time

In patients with sepsis and severe sepsis, dynamic changes in NETosis were assessed on days 1, 4, and 7 where permitted (see Figure 2(b)). Patients with sepsis (day 1: 45304 ± 11096 AFUs versus day 4: 45081 ± 11047 versus day 7: 44871 ± 19480; ANOVA, *p* = 0.99) and severe sepsis (day 1: 37181 ± 2204 AFUs versus day 4: 37513 ± 1954 AFUs versus day 7: 40116 ± 2622 AFUs, ANOVA, *p* = 0.68) did not show any significant change in NETosis over time. On day 4, NETosis in patients with severe sepsis (37513 ± 1954 AFUs, *N* = 39) was significantly lower compared to sepsis patients at day 4 (45081 ± 2017 AFUs, *N* = 30) and healthy controls at baseline (ANOVA, *p* = 0.001; see Figure 2(b)). By day 7, despite most patients with severe sepsis showing signs of sepsis resolution (SOFA 0-1), NETosis was persistently reduced compared to healthy controls (40116 ± 2622 AFUs versus 49659 ± 3285 AFUs; Student's *t*-test, *p* = 0.02; see Figure 2(b)). Neutrophils from nonresolving donors of sepsis by day 7 (SOFA > 3, *N* = 9) showed a trend towards generating a lower number of NETs compared with resolvers and healthy aged controls (39347 ± 6103 versus 42545 ± 2502 versus 49659 ± 3285), but this failed to reach significance (*p* = 0.17, ANOVA). To detect a significant difference, with 80% power (*p* = 0.05), a total of 29 patients with nonresolving sepsis would be required.

### 3.4. Suppressed NETosis Is Associated with Increased Early and Late Mortality

**Figure 3:**
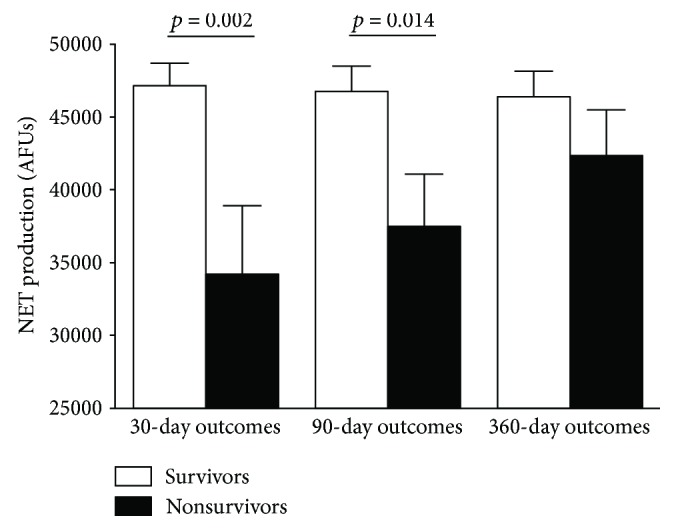
PMA-induced NET release is lower in nonsurvivors of sepsis at 30 days and 90 days. NET production in recruited patients on enrollment in response to PMA in survivors and nonsurvivors of sepsis at 30 days, 90 days, and 360 days following admission. Bars represent the mean with error bars the SEM, with *p* values from a Student's *t*-test.

Amongst all patients admitted with sepsis and severe sepsis, 16 died within 30 days of admission, with 80 surviving. Survivors of sepsis/severe sepsis had greater PMA-induced NETosis on admission than nonsurvivors had (47153 ± 1559 AFUs versus 34241 ± 4666 AFUs; Student's *t*-test *p* = 0.002; see Figure 3). By 90 days, mortality had risen to 24 patients and again was associated with an attenuated NETosis at admission to hospital (46772 ± 1744 versus 37498 ± 3602 AFUs; Student's *t* test, *p* = 0.014). There were no significant differences between survivors and nonsurvivors with regard to age at 30 days or 90 days.

The receiver operator curve (ROC) of survivors and nonsurvivors demonstrated an area under the curve of 0.70 (95% CI: 0.56–0.86; *p* = 0.011) with admission PMA-induced NET value of less than 39000 AFUs, having a 56% sensitivity and a 71% specificity for predicting 30-day mortality, whilst for 90-day mortality there was a 47% sensitivity and 70% specificity.

### 3.5. Suppression of NETosis Is Related to the Severity of Acidosis

**Figure 4:**
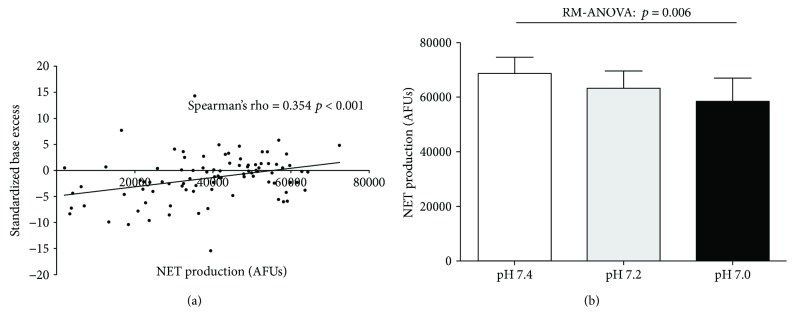
The impact of acidosis on neutrophil extracellular trap formation. (a) Correlation between standardized base excess and neutrophil extracellular trap formation in 99 patients with sepsis. Spearman's rho suggests that reduction in NET formation is associated with worsening acidosis. (b) Effect of acidosis on the NETs generated from the neutrophils of 5 healthy donors.

The standardized base excess (SBE) is a measure of metabolic acidosis. Patients with severe sepsis had a lower average SBE than those with sepsis had, who had a lower average SBE than healthy controls. Patients with greater deficits in measured SBE, reflecting a more severe metabolic acidosis, demonstrated a reduction in NETosis (Spearman rho = 0.348, 95% CI 0.147–0.521, *p* < 0.001) (see Figure 4). We therefore hypothesized that the acidosis induced by sepsis may alter neutrophil functions and be causally associated with the reduction of NETosis seen in patients with severe sepsis.

To investigate this, neutrophils from healthy donors were incubated in media (RPMI 1640) at a range of pathophysiological pHs (pH 7.4, 7.2, and 7.0) for 40 minutes prior to NETosis experiments being carried out as described previously. As the pH of the control media was reduced, there was a sequential fall in healthy donor neutrophils' ability to generate NETs in response to PMA stimulation (see Figure 4), although significance was only seen when comparing a pH of 7.4 with 7.0. Viability assays performed demonstrated no significant alteration in neutrophil viability induced by changes in pH at 4 hours.

### 3.6. Cell-Free DNA Is Raised in Patients with Sepsis

Circulating levels of plasma cf-DNA were measured on patients where sequential data points were available. Patients with sepsis (1308 ng/ml) and severe sepsis (1801 ng/ml) had significantly elevated levels of cf-DNA (ANOVA, *p* < 0.001) on admission compared to healthy controls (69 ng/ml) which persisted through to day 7 (ANOVA, *p* < 0.001) following admission (see online Supplementary Materials). No correlation was observed between NETosis and plasma cf-DNA (*p* = 0.988, Spearman's rho).

### 3.7. Severe Sepsis Causes Aberrant Neutrophil Migration

Chemotaxis (directional migration) towards CXCl-8 on admission in patients with severe sepsis (0.15 *μ*m/min, IQR 0.01–0.43 *μ*m/min) was significantly reduced compared to patients with sepsis (0.49 *μ*m/min, IQR 0.19–0.93 *μ*m/min) and healthy age-matched donor neutrophils (0.86 *μ*m/min {IQR 0.40–1.8 *μ*m/min}, Kruskal-Wallis; *p* < 0.001). Although patients with sepsis (without organ dysfunction) showed reduced chemotaxis compared to healthy controls, this failed to reach statistical significance (Dunn's; *p* = 0.15). Dysfunctional chemotaxis in severe sepsis patients (*N* = 32) persisted through to day 4 (0.35 *μ*m/min {IQR 0.09–0.85 *μ*m/min} versus 0.86 *μ*m/min {IQR 0.40–1.8 *μ*m/min}; Dunn's; *p* = 0.021). By day 7, chemotaxis in severe sepsis patients (*N* = 21) remained below levels seen in healthy elderly donors (0.60 *μ*m/min {IQR 0.30–1.1 *μ*m/min} versus 0.86 *μ*m/min {IQR 0.40–1.8 *μ*m/min}), but this failed to reach significance (Dunn's; *p* = 0.15).

### 3.8. Apoptosis Is Delayed in Neutrophils from Sepsis Patients

Neutrophil apoptosis from 18 patients with severe sepsis and 19 age-matched healthy controls was measured at the time of neutrophil isolation, at 4 hours and 24 hours postisolation. Patients with severe sepsis patients had a greater number of neutrophils in early (18.8% {IQR 13–32%} versus 5.3% {IQR 3.9–6.7%} *p* < 0.001, Mann–Whitney *U* test) and late apoptosis (2.8% {IQR 1.0–3.6%} versus 1.2% {IQR 0.5–2.1%} *p* = 0.03, Mann–Whitney *U* test) compared to healthy controls at the time of isolation.

At 4 hours, neutrophils from severe sepsis patients showed no difference in early and late apoptosis compared to baseline (early: 19.6% {IQR 15–33%} versus 18.8% {IQR 12–32%}, *p* = 0.19; late: 2.8% {1.0 versus 3.3% versus 2.6% {1.3–4.0} *p* = 0.45, Wilcoxon signed-rank tests), whilst healthy neutrophils showed significant increased early apoptosis 4 hours following isolation (10.2% {IQR 8–12%) versus 5.3% {IQR 3.6–5.8%}, Wilcoxon signed-rank test; *p* < 0.001).

At 24 hours following isolation, the rates of early (34.9 ± 22% versus 67.4 ± 15%, *p* < 0.001) and late apoptosis (9.4 ± 2.3% versus 19.8 ± 1.7%, *p* = 0.002) were significantly lower in severe sepsis patients compared with healthy patients, suggesting that neutrophil survival is prolonged during sepsis.

## 4. Discussion

This study investigated the sequential changes in neutrophil functions including NETosis and migration in a large cohort of sepsis and severe sepsis patients and related this to clinically relevant outcomes. Confirming our initial hypothesis, we demonstrated that severe sepsis is associated with a reduction in NETosis in systemic neutrophils which is not present in milder forms of sepsis. Furthermore, in severe sepsis, reduced NETosis persists through to days 4 and 7. Reduced NETosis was associated with important clinical outcomes including short- (30-day) and medium-term (90-day) mortality. To our knowledge, this is the largest study of NETosis in sepsis and the first that reports a relationship with impairment in innate immune cell function and patient survival. Suppression of ex vivo NETosis below 39000 AFUs was performed comparatively with other traditional biomarkers in predicting mortality from sepsis such as the severity of acidosis (SBE) and lactate [30].

We also observed a correlation between the severity of acidosis and the attenuation of NETosis in patients with sepsis. Furthermore, by manipulating the pH of the cellular environment in vitro, we could recapitulate the septic neutrophil phenotype suggesting that the disruption of cellular acid-base homeostasis may contribute to dysfunctional NETosis and perhaps other neutrophil functions. Changes in extracellular pH are common in many inflammatory diseases and lead to neutrophil activation, phagocytosis, and ROS production [31, 32]. NET formation in relation to pH has not been studied before, and these data add to the literature of the negative impact of acid-base disturbance.

Our data suggest that sepsis and the associated inflammation and changes in blood pH may induce a neutrophil phenotype characterised by poor migratory accuracy, reduced NETosis, and prolonged neutrophil survival. Previously, we have described aberrant neutrophil migratory accuracy with sepsis in pneumonia, but the current study is the first to study neutrophil functions and survival in such a large cohort of sepsis patients [12].

NETosis is a terminal event for neutrophils; thus, neutrophil activation and predominance of antiapoptotic pathways induced with sepsis may inhibit NETosis [31–33]. However, NETosis studies in patients with sepsis are hindered due to the failure to capture early NETosis at sepsis onset. Our study describes no difference in baseline NET release but reduced NET release following activation in patients with severe sepsis which does not recover at day 4 or 7 and a high burden of cf-DNA. These findings appear contradictory but are concordant with other studies of neutrophil function following a major host insult [34]. It has been proposed that the “first wave” of neutrophils to arrive at sites of infection is programmed for early NETosis in an attempt to contain the infection rapidly [35]. Those arriving later (which form the circulating pool on testing) may be activated by circulating cytokines and the septic environment and are resistant to apoptosis accounting for the attenuation of NETosis observed [32, 36].

The high levels of cell-free DNA would be consistent with evidence of an accumulation of NETs in inflammatory conditions caused by poor clearance [37]. Sepsis is associated with increased complement activity, and studies have shown that NETs activate the complement in vitro and deposited C1q inhibits NET degradation including a direct inhibition of DNase-I by C1q [38]. Clearance of nets may also be delayed due to suppression of DNases by renal/hepatic dysfunction present in multiorgan dysfunction [39]. This provides a potential mechanism for the presence of increased cell-free DNA with circulating neutrophils which are less able to undergo NETosis, as described in the current study and others [29, 40]. Cf-DNA is also gaining increasing attention, as a potential biomarker in sepsis, as it predicts outcomes from sepsis in patients admitted to intensive care and is relatively easy to measure [39, 41, 42]. However, more work is needed to determine the relationship of cf-DNA to cell functions and clinical outcomes.

The changes in neutrophil phenotype may drive further local and distant organ damage and predispose patients to secondary infections by inducing a state of immunosuppression [32, 43]. We propose a biphasic neutrophilic response to infection, with initial activation leading to migratory failure, frustrated phagocytosis, a release of large quantities of NETs, seen as cf-DNA, and a subsequent prolongation of neutrophil survival. The circulation of these free histones would activate the adaptive immune system, via dendritic cells, with the formation of anti-elastin, anti-histone, and anti-nuclear antibodies, as described in chronic inflammatory illnesses and autoimmune diseases, leading to further endothelial damage and exacerbating microcirculatory dysfunction [44, 45]. Sepsis-induced immunosuppression is also propagated by neutrophils that induce apoptosis in T-cells via the programmed death receptor and its ligand (PD-1/PD-L1) [8, 46, 47].

This study has limitations. Firstly, not all patients could have samples taken for analysis at all time points leading to the disparity in numbers across time points and assays. Patients with sepsis often experience a fluctuating course in their clinical recovery and require serial clinical blood tests, and missing time points are a common feature of studies of this nature [48, 49]. However, there were no differences in patient characteristics in all substudies to the main group, suggesting the data is representative of all patients.

Presepsis neutrophil function has not been assessed in these patients, and it is unclear whether preexisting neutrophil dysfunction is pathogenically associated with sepsis; it is possible that those with poorest outcomes had worse baseline neutrophil function. In survivors of the initial infectious insult, further neutrophil functions were not assessed beyond 7 days and so it is unclear if neutrophil function returns to levels expected in health following complete resolution or whether a permanent dysfunction results, predisposing these patients to further infectious insults.

A further limitation is the use of PMA as the stimulant to generate NETs. PMA is not physiological but causes maximal release of ROS and subsequently NETs by circumventing G receptor signaling [13]. This stimulus was chosen to assess maximal NET release, and similar concentrations have been used in several NET-related publications, enabling comparisons with published literature [13, 29, 40, 50].

## 5. Conclusions

Sepsis induces significant changes in neutrophil function. These may contribute to the failure of containment and the dissemination of the infection, whilst exaggerating the dysregulated immune response that is the hallmark of sepsis [4]. We propose that the combined dysfunctions result in a phenotypic immunoparesed neutrophil that contributes to the high mortality in patients with sepsis. Finally, we propose that further investigation into NETosis and cf-DNA as potential future biomarkers to identify high-risk patients with sepsis is warranted.

## Figures and Tables

**Table 1:** Characteristics of healthy controls and patients with sepsis and severe sepsis enrolled in the study.

	Healthy controls	Sepsis	Severe sepsis	Septic shock	*p* value
*N*	21	39	60	13	
Age	70	75	73	71	0.519^∗^
	(66–77)	(65–85)	(60–84)	(48–74)	
Sex, male (%)	13 (61.9)	20 (51.3)	41 (68.3)	10 (76.9)	0.070^#^
No. of comorbidities					
0	15	10	9	3	<0.001^Φ^
1	6	17	21	5	
2	0	7	19	3	
3+	0	5	11	2	
Comorbidities^§^, *n*					
None	15 (71)	10 (26)	9 (15)	3 (23)	0.076^Φ^
Hypertension	6 (29)	18 (46)	37 (62)	4 (31)	
IHD	0 (0)	10 (26)	15 (25)	2 (15)	
Asthma	0 (0)	0 (0)	0 (0)	1 (8)	
COPD	0 (0)	14 (36)	21 (35)	0 (0)	
Chronic renal disease	0 (0)	0 (0)	3 (5)	0 (0)	
Diabetes	0 (0)	3 (8)	17 (28)	4 (31)	
Medications^§^, *n*					
None	15	15	10	3	<0.001^Φ^
Antihypertensive	6	6	28	2	
Beta-blocker	1	8	16	3	
Antiplatelet	0	13	16	2	
Oral hypoglycemic	0	3	11	2	
Insulin	0	0	6	4	
Inhaled beta-agonist	0	6	16	0	
HMG-CoA reductase inhibitors	0	8	20	3	
APACHE II	N/A	14.5 (9–16)	16 (14–19)	19 (14–23)	<0.001
ICU Admission, *N*	N/A	0 (0%)	14 (23.3%)	13 (100%)	N/A
Vasopressors alone			2	2	
Respiratory support			4	3	
Multiorgan support			8	8	
WCC (10^9^/l)		14.8 (12.3–19.0)	15.1 (12.6–19.8)	20.4 (10.2–26.3)	0.634^∗∗^
CRP (mg/l)		153 (54.5–247)	100 (23.7–264)	132 (95–291)	0.232^∗∗^
Lactate (nM)	N/A	1.7 (1.1–2.1)	2.5 (2.0–3.4)	2.6 (1.3–4.1)	<0.001^∗∗^
SBE	N/A	0.2 (−1.2 to 2.6)	−2.2 (−5.6 to 0.5)	−4.0 (−7.2 to −2.2)	0.001^∗∗^
SOFA score	N/A	1 (0-1)	3 (1–6)	9 (6–12)	<0.001^∗∗^
Length of stay, days	N/A	10 (6–19)	10 (6.3–19.8)	21.5 (10–67)	0.505^∗∗^
Mortality, *n* (%)					
30-day	0	4 (10)	12 (20)	7 (53)	0.265^#^
90-day	0	7 (18)	17 (28)	8 (62)	0.337^#^
360-day	0	11 (28)	22 (37)	8 (62)	0.513^#^

The baseline characteristics of healthy participants and patients recruited. The septic shock cohort is a subset of the patients with severe sepsis. The *p* values have been calculated using healthy, sepsis, and severe sepsis participant cohorts where appropriate. *p* values represented by ∗ are from a Kruskal-Wallis test, # from a Fisher's exact test, Φ from a *χ* ^2^ test, and ∗∗ from a Mann–Whitney *U* test.

## Data Availability

The datasets used and/or analysed during the current study are available from the corresponding author on reasonable request.

## Abbreviations

AFUs: Arbitrary fluorescent units
ANOVA: Analysis of variance
cf-DNA: Cell-free DNA
CXCL-8: Interleukin-8
GPS: Glutamine, penicillin, and streptomycin
IQR: Interquartile range
NET: Neutrophil extracellular trap
PD-1: Programmed death receptor-1
PMA: Phorbol-myristate acetate
ROS: Reactive oxygen species
SBE: Standardized base excess
SEM: Standard error of the mean
USA: United States of America.

## Supplementary Materials

Supplementary Materials Table 1: criteria used for the definition of patients with sepsis and severe sepsis. Table 2: cell-free DNA values in healthy controls and sepsis and severe sepsis patients at days 1 and 7. Figure 1: severe sepsis is associated with reduced chemotaxis. (A) The difference in chemotaxis between healthy aged controls, sepsis patients, and severe sepsis patients on admission to hospital. (B) Sequential changes in neutrophil chemotaxis in patients with sepsis (day 1 *N*=28, day 4 *N*=18, and day 7 *N*=14) and severe sepsis (day 1 *N*=42, day 4 *N*=32, and day 7 *N*=21). Bars represent the median and IQR with the error bars from Tukey's distribution. *p* values from a Kruskal-Wallis test.

## References

[1] Angus D. C., Linde-Zwirble W. T., Lidicker J., Clermont G., Carcillo J., Pinsky M. R. (2001). Epidemiology of severe sepsis in the United States: analysis of incidence, outcome, and associated costs of care. *Critical Care Medicine*.

[2] Angus D. C., van der Poll T. (2013). Severe sepsis and septic shock. *The New England Journal of Medicine*.

[3] Adhikari N. K. J., Fowler R. A., Bhagwanjee S., Rubenfeld G. D. (2010). Critical care and the global burden of critical illness in adults. *Lancet*.

[4] Singer M., Deutschman C. S., Seymour C. W. (2016). The Third International Consensus Definitions for Sepsis and Septic Shock (Sepsis-3). *JAMA*.

[5] Hotchkiss R. S., Karl I. E. (2003). The pathophysiology and treatment of sepsis. *The New England Journal of Medicine*.

[6] Borregaard N. (2010). Neutrophils, from marrow to microbes. *Immunity*.

[7] Brown K. A., Brain S. D., Pearson J. D., Edgeworth J. D., Lewis S. M., Treacher D. F. (2006). Neutrophils in development of multiple organ failure in sepsis. *Lancet*.

[8] Leliefeld P. H. C., Wessels C. M., Leenen L. P. H., Koenderman L., Pillay J. (2016). The role of neutrophils in immune dysfunction during severe inflammation. *Critical Care*.

[9] Alves-Filho J. C., de Freitas A., Spiller F., Souto F. O., Cunha F. Q. (2008). The role of neutrophils in severe sepsis. *Shock*.

[10] Delano M. J., Thayer T., Gabrilovich S. (2011). Sepsis induces early alterations in innate immunity that impact mortality to secondary infection. *Journal of Immunology*.

[11] Fialkow L., Fochesatto Filho L., Bozzetti M. C. (2006). Neutrophil apoptosis: a marker of disease severity in sepsis and sepsis-induced acute respiratory distress syndrome. *Critical Care*.

[12] Sapey E., Patel J. M., Greenwood H. L. (2017). Pulmonary infections in the elderly lead to impaired neutrophil targeting, which is improved by simvastatin. *American Journal of Respiratory and Critical Care Medicine*.

[13] Brinkmann V., Reichard U., Goosmann C. (2004). Neutrophil extracellular traps kill bacteria. *Science*.

[14] Landoni V. I., Chiarella P., Martire-Greco D. (2012). Tolerance to lipopolysaccharide promotes an enhanced neutrophil extracellular traps formation leading to a more efficient bacterial clearance in mice. *Clinical and Experimental Immunology*.

[15] Li P., Li M., Lindberg M. R., Kennett M. J., Xiong N., Wang Y. (2010). PAD4 is essential for antibacterial innate immunity mediated by neutrophil extracellular traps. *The Journal of Experimental Medicine*.

[16] Arazna M., Pruchniak M. P., Demkow U. (2013). Neutrophil extracellular traps in bacterial infections: strategies for escaping from killing. *Respiratory Physiology & Neurobiology*.

[17] Meng W., Paunel-Gorgulu A., Flohe S. (2012). Depletion of neutrophil extracellular traps in vivo results in hypersusceptibility to polymicrobial sepsis in mice. *Critical Care*.

[18] Narasaraju T., Yang E., Samy R. P. (2011). Excessive neutrophils and neutrophil extracellular traps contribute to acute lung injury of influenza pneumonitis. *The American Journal of Pathology*.

[19] Clark S. R., Ma A. C., Tavener S. A. (2007). Platelet TLR4 activates neutrophil extracellular traps to ensnare bacteria in septic blood. *Nature Medicine*.

[20] Gardiner E. E., Andrews R. K. (2012). Neutrophil extracellular traps (NETs) and infection-related vascular dysfunction. *Blood Reviews*.

[21] Thomas G. M., Carbo C., Curtis B. R. (2012). Extracellular DNA traps are associated with the pathogenesis of TRALI in humans and mice. *Blood*.

[22] Villanueva E., Yalavarthi S., Berthier C. C. (2011). Netting neutrophils induce endothelial damage, infiltrate tissues, and expose immunostimulatory molecules in systemic lupus erythematosus. *Journal of Immunology*.

[23] Dellinger R. P., Levy M. M., Carlet J. M. (2008). American Association of Critical-Care Nurses, American College of Chest Physicians, American College of Emergency Physicians., Canadian Critical Care Society., European Society of Clinical Microbiology and Infectious Diseases., European Society of Intensive Care Medicine., European Respiratory Society., International Sepsis Forum., Japanese Association for Acute Medicine., Japanese Society of Intensive Care Medicine., Society of Critical Care Medicine., Society of Hospital Medicine., Surgical Infection Society., World Federation of Societies of Intensive and Critical Care Medicine. Surviving sepsis campaign: international guidelines for management of severe sepsis and septic shock: 2008. *Critical Care Medicine*.

[24] Butcher S. K., Chahal H., Nayak L. (2001). Senescence in innate immune responses: reduced neutrophil phagocytic capacity and CD16 expression in elderly humans. *Journal of Leukocyte Biology*.

[25] Sapey E., Greenwood H., Walton G. (2014). Phosphoinositide 3-kinase inhibition restores neutrophil accuracy in the elderly: toward targeted treatments for immunosenescence. *Blood*.

[26] Hazeldine J., Harris P., Chapple I. L. (2014). Impaired neutrophil extracellular trap formation: a novel defect in the innate immune system of aged individuals. *Aging Cell*.

[27] Andrew N., Insall R. H. (2007). Chemotaxis in shallow gradients is mediated independently of PtdIns 3-kinase by biased choices between random protrusions. *Nature Cell Biology*.

[28] Sapey E., Stockley J. A., Greenwood H. (2011). Behavioral and structural differences in migrating peripheral neutrophils from patients with chronic obstructive pulmonary disease. *American Journal of Respiratory and Critical Care Medicine*.

[29] Hampson P., Dinsdale R. J., Wearn C. M. (2017). Neutrophil dysfunction, immature granulocytes, and cell-free DNA are early biomarkers of sepsis in burn-injured patients; a prospective observational cohort study. *Annals of Surgery*.

[30] Smith I., Kumar P., Molloy S. (2001). Base excess and lactate as prognostic indicators for patients admitted to intensive care. *Intensive Care Medicine*.

[31] Trevani A. S., Andonegui G., Giordano M. (1999). Extracellular acidification induces human neutrophil activation. *Journal of Immunology*.

[32] Diaz F. E., Dantas E., Cabrera M. (2016). Fever-range hyperthermia improves the anti-apoptotic effect induced by low pH on human neutrophils promoting a proangiogenic profile. *Cell Death & Disease*.

[33] Wann J. G., Hsu Y. H., Yang C. C. (2007). Neutrophils in acidotic haemodialysed patients have lower intracellular pH and inflamed state. *Nephrology, Dialysis, Transplantation*.

[34] Hazeldine J., Naumann D. N., Toman E. (2017). Prehospital immune responses and development of multiple organ dysfunction syndrome following traumatic injury: a prospective cohort study. *PLoS Medicine*.

[35] Yipp B. G., Kubes P. (2013). NETosis: how vital is it?. *Blood*.

[36] Drifte G., Dunn-Siegrist I., Tissieres P., Pugin J. (2013). Innate immune functions of immature neutrophils in patients with sepsis and severe systemic inflammatory response syndrome. *Critical Care Medicine*.

[37] Mahajan A., Herrmann M., Munoz L. E. (2016). Clearance deficiency and cell death pathways: a model for the pathogenesis of SLE. *Frontiers in Immunology*.

[38] Leffler J., Martin M., Gullstrand B. (2012). Neutrophil extracellular traps that are not degraded in systemic lupus erythematosus activate complement exacerbating the disease. *Journal of Immunology*.

[39] Saukkonen K., Lakkisto P., Pettila V. (2008). Cell-free plasma DNA as a predictor of outcome in severe sepsis and septic shock. *Clinical Chemistry*.

[40] Hashiba M., Huq A., Tomino A. (2015). Neutrophil extracellular traps in patients with sepsis. *The Journal of Surgical Research*.

[41] Huttunen R., Kuparinen T., Jylhava J. (2011). Fatal outcome in bacteremia is characterized by high plasma cell free DNA concentration and apoptotic DNA fragmentation: a prospective cohort study. *PLoS One*.

[42] Margraf S., Logters T., Reipen J., Altrichter J., Scholz M., Windolf J. (2008). Neutrophil-derived circulating free DNA (cf-DNA/NETs): a potential prognostic marker for posttraumatic development of inflammatory second hit and sepsis. *Shock*.

[43] Juss J. K., House D., Amour A. (2016). Acute respiratory distress syndrome neutrophils have a distinct phenotype and are resistant to phosphoinositide 3-kinase inhibition. *American Journal of Respiratory and Critical Care Medicine*.

[44] Diamantopoulos A. P. (2013). Extracellular neutrophil traps: a novel therapeutic target in ANCA-associated vasculitis?. *Frontiers in Immunology*.

[45] Tillack K., Breiden P., Martin R., Sospedra M. (2012). T lymphocyte priming by neutrophil extracellular traps links innate and adaptive immune responses. *Journal of Immunology*.

[46] Demaret J., Venet F., Friggeri A. (2015). Marked alterations of neutrophil functions during sepsis-induced immunosuppression. *Journal of Leukocyte Biology*.

[47] Patera A. C., Drewry A. M., Chang K., Beiter E. R., Osborne D., Hotchkiss R. S. (2016). Frontline science: defects in immune function in patients with sepsis are associated with PD-1 or PD-L1 expression and can be restored by antibodies targeting PD-1 or PD-L1. *Journal of Leukocyte Biology*.

[48] Patel J. M., Snaith C., Thickett D. R. (2012). Randomized double-blind placebo-controlled trial of 40 mg/day of atorvastatin in reducing the severity of sepsis in ward patients (ASEPSIS Trial). *Critical Care*.

[49] Craig T. R., Duffy M. J., Shyamsundar M. (2011). A randomized clinical trial of hydroxymethylglutaryl-coenzyme a reductase inhibition for acute lung injury (the HARP Study). *American Journal of Respiratory and Critical Care Medicine*.

[50] Brinkmann V., Laube B., Abu Abed U., Goosmann C., Zychlinsky A. (2010). Neutrophil extracellular traps: how to generate and visualize them. *Journal of Visualized Experiments*.

