# Supplementary material for: Sepsis Induces a Dysregulated Neutrophil Phenotype That Is Associated with Increased Mortality
Source: Mediators Inflamm. 2018 Apr 11;2018:4065362. doi: 10.1155/2018/4065362 (PMC5925119; doi:10.1155/2018/4065362)
Supplement: Supplementary Materials — Table 1: criteria used for the definition of patients with sepsis and severe sepsis. Table 2: cell-free DNA values in healthy controls and sepsis and severe sepsis patients at days 1 and 7. Figure 1: severe sepsis is associated with reduced chemotaxis. (A) The difference in chemotaxis between healthy aged controls, sepsis patients, and severe sepsis patients on admission to hospital. (B) Sequential changes in neutrophil chemotaxis in patients with sepsis (day 1 N=28, day 4 N=18, and day 7 N=14) and severe sepsis (day 1 N=42, day 4 N=32, and day 7 N=21). Bars represent the median and IQR with the error bars from Tukey's distribution. p values from a Kruskal-Wallis test. [file 4065362.f1.pdf]

Online supplement

Table 1: Criteria used for the definition of patients with Sepsis and Severe Sepsis.

**SIRS**	2 or more of the following criteria:<br>Fever of more than 38.3°C or less than 36°C<br>Heart rate of more than 90 beats per minute<br>Respiratory rate of more than 20 breaths per minute or a $PaCO_2$ level of less than 32 mm Hg<br>Abnormal white blood cell count (>12,000/μL or <4,000/μL or >10% bands)
**Sepsis**	SIRS + new/suspected infection<br>S
**Severe Sepsis**	Sepsis + sepsis-induced organ dysfunction
Organ Dysfunction	*Sepsis-induced hypotension*<br>*Lactate > normal laboratory results*<br>*Urine output <0.5 mL/kg hr. for >2 hrs.,*<br>*ALI with PaO_2/FIO_2 <250 in the absence of pneumonia as infection source*<br>*ALI with PaO_2/FIO_2 <200 in the presence of pneumonia as infection source*<br>*Creatinine >176.8 mmol/L)*<br>*Bilirubin >34.2 mmol/L)*<br>*Platelet count >100,000/mm^3*<br>*Coagulopathy (INR>1.5)*
Septic Shock	*Severe sepsis + hypotension not reversed by fluid resuscitation*

SIRS: systemic inflammatory response syndrome

ALI: Acute lung injury

Additional Methods

Neutrophil Isolation

Neutrophils were isolated on a Percoll (pH 8.5-9.5; Sigma-Aldrich, Dorset, UK) density gradient as previously described (87). Blood collected in Lithium Heparin and EDTA vacutainers (Becton Dickinson) were transferred into separate 50ml sterile Falcon^{TM} tubes (Becton Dickinson). 2% dextran (Sigma-Aldrich) was added (1ml for every 6mls of blood) and gently mixed prior to incubation for 30minutes at room temperature to sediment the erythrocytes. The leucocyte-rich plasma was carefully layered on a Percoll (Sigma-Aldrich) density gradient consisting of 2.5mls of 80% Percoll (Sigma-Aldrich) and 5 mls of 56% Percoll (Sigma-Aldrich) (see figure 2.1).

To prepare the gradients, a working stock of Percoll was made by mixing 45 ml of Percoll (Sigma-Aldrich) with 5 ml of 9% (v/v) sodium chloride (Sigma-Aldrich). The 80% Percoll was prepared by diluting 40 ml of the Percoll stock with 10 ml 0.9% (v/v) sodium chloride (Baxter) whilst 56% Percoll comprised of 28 ml of the working Percoll stock and 22 ml of 0.9% (v/v) sodium chloride (Baxter). Gradients were then prepared by carefully layering 5 ml of 56% Percoll on top of 2.5 ml 80% Percoll in a 15ml sterile Falcon^{TM} tube (Becton Dickinson).

The gradients and leucocyte-rich plasma were centrifuged at 220G for 20 minutes with no acceleration and no brake at room temperature. Neutrophils were removed from the 80% and 56% gradient interface and re-suspended then washed in phosphate buffered saline (PBS; Gibco Invitrogen, Paisley, UK) at 440G for 10minutes at room temperature. Post-centrifugation the

supernatant was discarded and the cells re-suspended in either RPMI 1640 (Sigma-Aldrich) or appropriate media. Purity was assessed by cytospin and staining with Giemsa stain (Diff-Qik; Gentaur Europe, Brussels, Belgium) routinely yielding neutrophil purity of greater than 95% and a viability of >98% as evaluated by tryptan blue exclusion

Neutrophil Migration

Neutrophil migration was assessed using an Insall Chamber (Weber Scientific International Ltd, Teddington). This improved chemotaxis chamber allows the migratory dynamics of individual cells to be assessed and provides greater information than traditional methods that provide only quantitative measures of cell migration.

Isolated neutrophils from Lithium-Heparin vacutainers were re-suspended in RPMI 1640 (Sigma-Aldrich) at a concentration of 5 $x10^6$/ml. Bovine Albumin Fraction V (Sigma-Aldrich) was added to the neutrophils at a final concentration of 1.125% v/v. Neutrophils were then placed on a cleaned (with 0.4M H_2SO_4) albumin coated coverslip (22x22mm, Surgipath Medical Industries Inc. Europe) and allowed to adhere for 20 minutes at room temperature. Once adhered the coverslip was inverted and placed on a clean Insall Chamber pre-filled with sterile RPMI 1640 (Sigma-Aldrich). The RPMI 1640 (Sigma-Aldrich) was replaced with 100nM CXCL-8 (R&D systems, Abingdon, UK) as chemoattractants. Gradients were allowed to develop for 5 minutes before assessment of migration.

Real-time video microscopy using a Leica DMI6000B with DFC360FX camera was used to capture neutrophil migration. Images were captured every 20 seconds for 12minutes producing 36 frames. This time frame was considered optimal for migration studies. Images were analysed using Image J software (Wayne Rasband, NIH, Bethesda) using vector analysis. Images were divided into ten individual segments and one cell randomly selected within each segment for analysis. Therefore, ten randomly selected cells were analysed.

NETosis Experiments

Freshly isolated neutrophils from EDTA vacutainers (Becton Dickinson) were used in the quantification of NET formation. Neutrophils were re-suspended in RPMI 1640 (Sigma-Aldrich) supplemented with 2nM L-Glutamine, 100U/ml Streptomycin and 100ug/ml Penicillin (GPS; all purchased from Sigma-Aldrich), to ensure the culture remained sterile, at a concentration of 1 $x10^6$/ml. 1 $x10^5$ cells were placed in a 96-well flat bottomed plate (Becton Dickinson) with an additional 75µl of RPMI 1640 with GPS (Sigma-Aldrich). Cells were then stimulated with 25nM PMA (Sigma-Aldrich) or an additional 25µl of the media was added for the negative control and incubated for 3 hours at 37°C supplemented with 5% CO_2. Experiments were performed in quadruplicate.

Following incubation samples were treated with 200units of micrococcal nuclease (MNase; Sigma-Aldrich) and 1µM of SYTOX Green (Invitrogen) and incubated in the dark for 10 minutes at room temperature. This process stained and digested the extracellular DNA only. Samples were then transferred to into 500µl eppindorfs and pelleted at 5000rpm for 10minutes. The DNA containing

supernatant was transferred (170μl) into a 96- well black flat-bottomed plate (CoStar; Sigma-Aldrich) and fluorescence measured in a BioTek Synergy 2 fluorometric plate reader (NorthStar Scientific Ltd, Leeds, UK) with a filter setting of 485nm excitation and 530nm emission. Background fluorescence from unstimulated negative controls was subtracted from the stimulated values.

Neutrophil Apoptosis

Freshly isolated neutrophils from EDTA vacutainers (Becton Dickinson) were suspended in RPMI 1640 with GPS (Sigma-Aldrich) at a concentration of $1x10^6$/ml. $2x10^5$ cells were added to cytometric tubes and the cells pelleted by centrifugation at 600G for 4minutes at 4°C. The pellet was washed twice and re-suspended (600G for 4minutes at 4°C) in Annexin buffer (BD Biosciences, Oxford, UK).

Apoptosis was assessed by flow cytometer using a $CyAN_{ADP}$ (Beckman Coulter). The washed cells were stained with Fluorescein Isothiocyanate (FITC) conjugated Annexin V (dilution 1:100; BD Biosciences) and incubated for 20 minutes at 4°C. Just prior to flow cytometric analysis SYTOX Blue (conjugated to violet, dilution 1:2000; Invitrogen) was added to the samples. Cell debris was eliminated using appropriate side and forward scatter gating. Data was analysed using Summit v.4.3. (Dako). The software carried out colour compensation automatically. Areas of cells that were negative for both (alive) Annexin V only positive (early apoptotic), positive for both (late apoptotic) and

SYTOX Blue only positive (necrotic) were individually calculated. Data is represented as the percentage of cells in various stages of apoptosis.

Table 2: Cell-free DNA values in Healthy controls, sepsis and severe sepsis patients at day 1 and 7

	Healthy Controls	Sepsis	Severe Sepsis	p-value
**Cf-DNA Day 1 (ng/ml)**	68.6 (42.1-111.5)	1308 (1246-1636)	1826 (1426-2241)	<0.0001
**Cf-DNA Day 7 (ng/ml)**		1333 (1160-1690)	1608 (1066-2072)	<0.0001

The table represents the cell-free (Cf) DNA from healthy controls and patients with sepsis and severe sepsis on days 1 and 7. Values represent the median (IQR) and p-values are from a Kruskal-Wallis test.

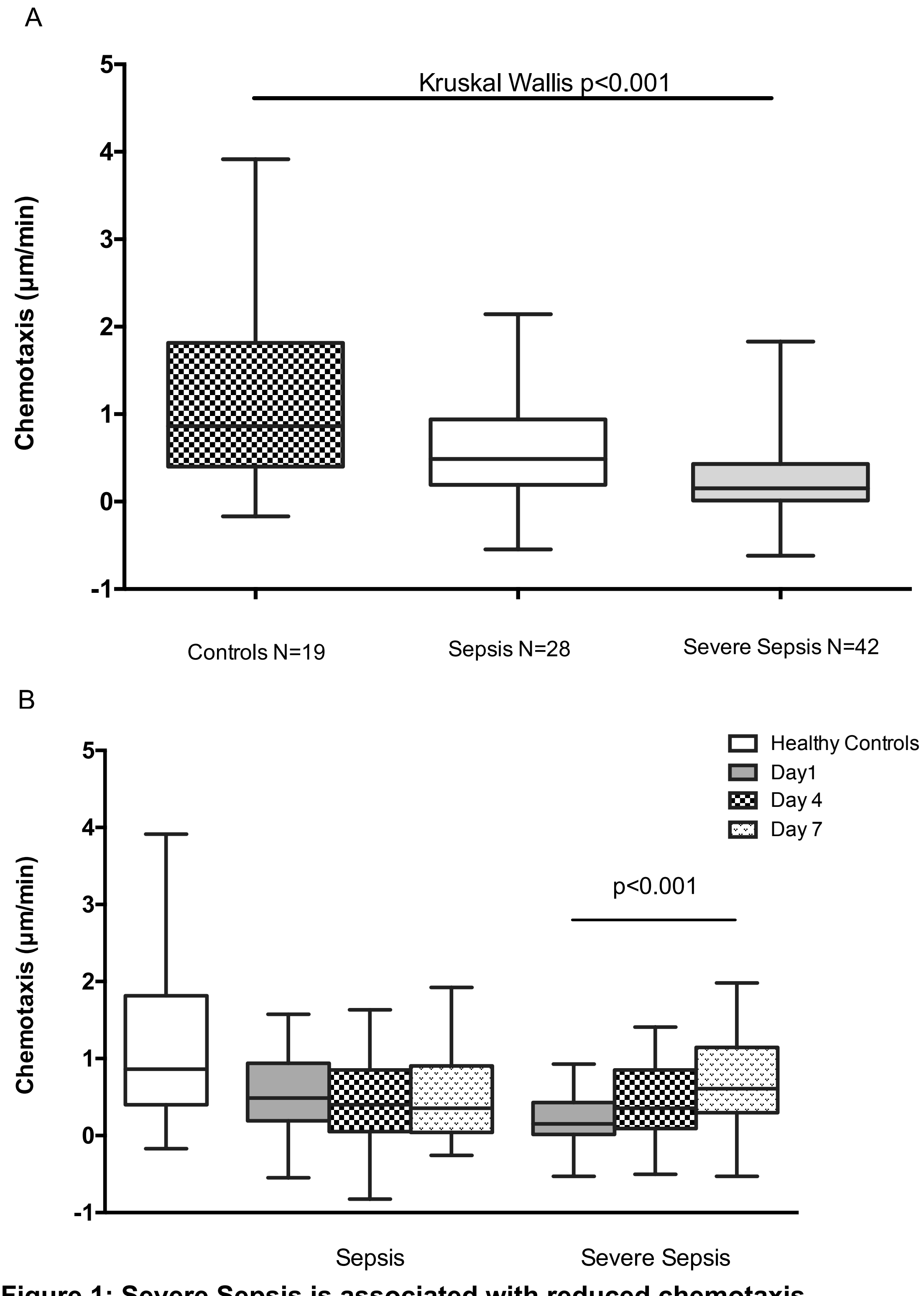

Figure 1: Severe Sepsis is associated with reduced chemotaxis

A: The difference in chemotaxis between healthy aged controls, sepsis patients and severe sepsis patient on admission to hospital. B: the sequential changes in neutrophil chemotaxis in patients with sepsis (Day 1=28, day 4 N=18 and day 7 N=14) and severe sepsis (day 1 N=42, day 4 N=32 and day 7 N=21). Bars represent the median and IQR with the error bars from a Tukey's distribution. P-values from a Kruskal-Wallis test.
